# Analysis of the Fire Properties of Blown Insulation from Crushed Straw in the Buildings

**DOI:** 10.3390/ma14154336

**Published:** 2021-08-03

**Authors:** Jiří Teslík

**Affiliations:** Department of Building Constructions, Faculty of Civil Engineering, VSB-Technical University of Ostrava, Ludvíka Podéště 1875/17, 70833 Ostrava-Poruba, Czech Republic; jiri.teslik@vsb.cz

**Keywords:** crushed straw, blown insulation, fire test, sustainability, fire reaction class, civil engineering

## Abstract

Sustainable development in civil engineering is the clear and necessary goal of the current generation. There are many possibilities for reducing the use of depletable resources. One of them is to use renewable and recyclable materials on a larger scale in the construction industry. One possibility is the application of natural thermal insulators. A typical example is a crushed straw, which is generated as agricultural waste in the Czech Republic. Due to its small dimensions and good thermal insulation parameters, this material can also be used as blown thermal insulation. The research aims to examine the fire resistance of crushed straw as blown insulation. The single-flame source fire test results, thermal attack by a single burning item (SBI) test and large-scale test of a perimeter wall segment are shown. The results show that blown insulation made of crushed straw meets the requirements of fire protection. In addition, crushed straw can be also used to protect load-bearing structures due to its behaviour. This article also shows the production process of crushed straw used as blown insulation in brief.

## 1. Introduction

Sustainable development in civil engineering entails not only the need to improve conventional construction methods and materials but also the development of new methods or rediscovering forgotten techniques [1,2]. This is also a larger problem at this time, as building material prices are rising. Straw as a part of buildings (roofs, insulation and load-bearing elements) appeared in the Middle Ages and was also used at the beginning of the 20th century [3]. The use of straw in construction complies with the principles of sustainable development thanks to the use of the material as a secondary raw material [4,5].

In the Czech Republic, 2 million tonnes of straw are wasted annually [6]. Worldwide, a large amount of straw is burned on the field [7] or used in incinerators for energy production [8], which entails a certain increase in the environmental burden. However, it can be suppressed by alternative uses of straw. We must not forget the increased interest in ecological construction due to the indisputable need to further reduce the impact of human activities on the environment and global climate problems [9,10,11].

Straw can be used as prefabricated straw wall panels [12,13] or self-supporting straw bales [14,15]. Another possible use of straw in construction can be various composite structural elements, for example in the form of a straw fibre cement composite [16,17]. A very interesting possibility is the application of crushed straw in construction as blown insulation [18]. It should be noted that each type of use of straw has a different economic and environmental impact and especially different principles of behaviour of the entire structure and individual elements.

The thermal insulation [17,19,20], sound [21,22], moisture [23,24] and diffusion [25] properties of straw are relatively well researched, but there is a lack of more extensive knowledge of fire resistance, flammability and fire parameters. Fire characteristics of building materials hold an important role in building design [26]. This aspect is even more important for buildings made of natural materials than, for example, for masonry buildings, as natural materials are expected to be less resistant to fire than industrially produced materials. Additionally, information about less typical materials and structures and resistance to thermal attack is available, which can be used in fire resistance research [27,28,29]. This information can be considered in the research of crushed straw fire resistance. A lot of information about fire tests of straw bales can be found [3,30,31,32], but there is less information about blown insulation with crushed straw. The article builds on previous research [33,34] and complements it with new knowledge, especially in the field of evaluation of large-format fire tests. The research aims to prove the fire resistance of crushed straw as blown insulation.

## 2. Production and Application of Crushed Straw as Insulation

### 2.1. Production

**Figure 1:**
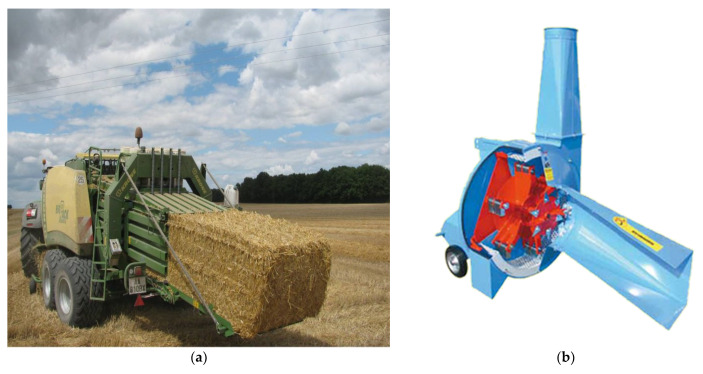
Harvesting press for large prismatic straw bales (**a**); biomass shredder (**b**) [36].

The production process of crushed straw begins with the harvest of cereals [35]. By mowing grown cereals, loose straw stalks are created and can also be tied into bales. More often, however, straw blades are left in the field, where they dry out to a moisture value of 15% under favourable climatic conditions. Then, the loose stalks are removed using a baling machine (see Figure 1a) [36], which creates bales from them. The compression and shape of the product depend on the type of the machine, which produces bales in the form of rollers or blocks. After creating bales in the baling machine, where the stalks are tied together at the same time, the bales are transported to covered warehouses for storage.

The next step leading to the formation of crushed straw is the actual crushing of the straw stalks in the tied bales. After transporting the bales from the warehouse, the bales are placed on a receiving table or a rake conveyor. These machines transport the bales to a shredder (see Figure 1b) [36]. The disassembled stalks are then cut to a length of about 150 mm. The cut stalks are then crushed to the desired fraction in a hammer crusher. The crushed straw is then often used as bedding for livestock.

### 2.2. Preparation and Application 

**Figure 2:**
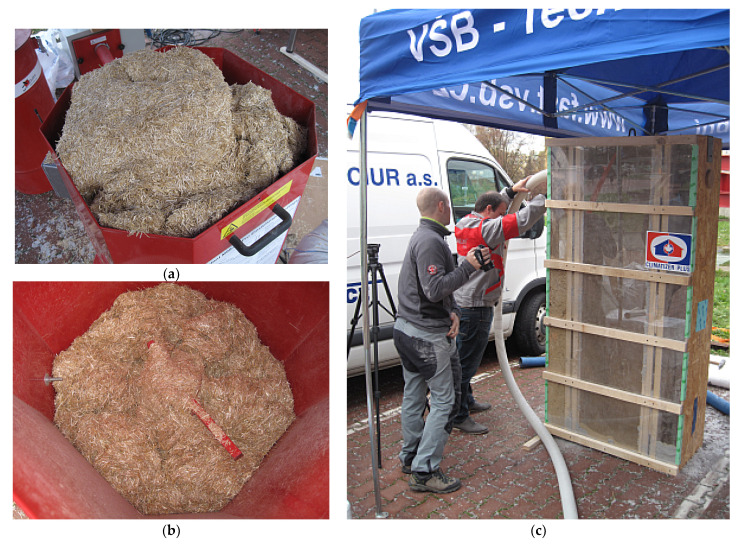
Crushed straw placed in the application machine (**a**); the lower part of the application machine with rotating blades (**b**); an example of application of crushed straw to the test segment (**c**).

Blown insulation is an alternative to the insulation available today in the form of mats or boards. Blown insulation made of cellulose or wooden fibres is known and popular. In the future, crushed straw can be among the standard natural blown insulations. In contrast, the main advantage is the application of the straw by using a blower; therefore, they can fill even hard-to-reach places and spaces. In the implementation, crushed straw is poured into the application machine (see Figure 2a), and the insulator is divided into small parts using rotary blades (see Figure 2b). Subsequently, the insulation is transported to the destination through air pressure and hose lines. The application machine can be set exactly on the insulation material parameters. Because of this setting, the optimal bulk density of the insulation in the structure can be obtained to prevent it from settling [18]. Another advantage of this type of insulation, compared to boards and mats, is the minimisation of waste during application. 

## 3. Fire Tests of Crushed Straw

Not only in the Czech Republic is the fire resistance determined in minutes according to legislation [37,38]. For the evaluation of materials, the reaction to fire class is used. This class shows how resistant a given material is to the spread and development of fire. Other criteria for classification into reaction to fire are the amount of energy released by the material during a fire, the intensity of smoke during the burning of the material and the occurrence of burning drops. This classification is based on tests performed in certified laboratories according to the procedure prescribed by legislation [37,38]. 

### 3.1. Single-Flame Source Fire Test 

This experiment was partially introduced before in [33], but because the single-flame source test was the first step in determining the fire resistance, the basic principle and results are described here as well. The test was conducted according to EN ISO 11925-2 [39]. A vertical container with a minimum dimension of 180 mm × 90 mm × 40 mm was prepared and filled with the tested crushed straw. The test container was made of wire mesh, and on the exposed site was the opening for the flame. A burner with a small standard flame was placed at an angle of 45° at a distance of 40 mm above the lower edge of the container. The test was performed by moving the burner onto the test specimen at such a distance that its flame touched the specified point of contact. The burner remained in this position for 15 s. The test was performed on five samples. After the test, the sample was evaluated based on its ignition and whether the burnout reached a height of 150 mm from the point of contact.

### 3.2. Thermal Attack by a Single Burning Item (SBI) Fire Test

To determine the reaction to fire classes A2 to D, thermal attack by a single burning item (SBI) according to EN 13823 was used [40]. This test simulates the course of a fire in a corner of a room on a real scale. The tested material was prepared in a vertical form in a room with floor plan dimensions of 3 m × 3 m and a height of 2.4 m. The unexposed surface of the test specimen was made of oriented strand board (OSB) panels. Filling holes for crushed straw application were located at the top of the samples. The fire source was set at a critical point (corner) on the floor.

The test segment consisted of two parts 0.5 m and 1.0 m wide. Both parts were 1.5 m high. A 30 kW propane sand burner with at least 95% technical propane technology was used to cause a fire. A flue gas extractor was set up in the upper part of the room. The classification parameters of the SBI test are:

- Fire growth rate (FIGRA) index;
- Total heat release (THR600s);
- Smoke production as smoke growth rate (SMOGRA) index;
- Total smoke production (TSP600s);
- Lateral flame spread (LFS);
- Flaming droplets and particles according to their occurrence during the first 600 s of the test. After measuring and calculating, these values were used to classify the reaction to fire class according to the criteria given in Table 1.

### 3.3. Large-Scale Fire Test of a Wall Segment

Large-scale fire tests are most often used to verify the fire resistance parameters of entire building structures. Real structures are exposed to fire tests, which are performed in specialised and certified laboratories equipped with the necessary technical facility for these tests. In addition to the large-format fire test, which is expensive, a cheaper variant can be used for the preliminary determination and verification of fire resistance with the possibility of verifying several test specimens at once. This cheaper variant is called a preliminary fire test according to EN 1364-1 [41]. In contrast to the classical test, the segment is tested for informative purposes with smaller dimensions, only 0.8 m × 0.8 m, and the test cannot be performed with the loading of the samples because the individual segment is separated from each other by a lining. This test can be used to determine the value of fire resistance *I* (insulation) (min) and *E* (integrity) (min). It is not possible to determine the value of fire resistance *R* (load capacity) (min).

For the analysis of different combinations of properties, three different variants of the cladding were prepared together with crushed straw (see Table 2). The supporting structure of the test specimens was made of a vertically perforated LAG frame [42]. The test specimens were sheathed and filled with crushed straw with a bulk density of 90 kg·m^−3^. 

This test aimed to determine the value of fire resistance *EI* (min) of the tested samples. The value of *E* (integrity) indicates the ability of the material (sample) to prevent the passage of flame and hot gases when heated on one side and to prevent the occurrence of flames on the unexposed side. The value *I* (insulation) indicates the ability of the material (sample) to limit the temperature rise on the other side when heating one side [41]. The criteria for cracks, holes and continuous combustion are checked visually with gauges. For criterion *I*, the decisive factor is the time during which the temperature on the unexposed surface of the sample rises by 140 °C from the average initial temperature of this surface. The temperature is measured using thermocouples during the test. 

To ensure the external conditions for the preliminary fire test according to the standard [41], it was necessary to prepare the samples under such conditions that the moisture content of the sample was close to the normal conditions in practice. During the test, the ambient air temperature in the vicinity of the test furnace must not fall by more than 10 °C and must not increase by more than 20 °C from an initial temperature of between 10 °C and 40 °C. It is also important to check the oven temperature, which must not rise above 50 °C for five minutes before starting the test. The values recorded by the individual thermoelectric sensors before the test needs to be checked. These values determine the initial measurement temperature. The test time is recorded from the moment the burners ignite in the furnace. The sensors record the temperature every minute.

## 4. Results

### 4.1. Single-Flame Source Fire Test

**Figure 3:**
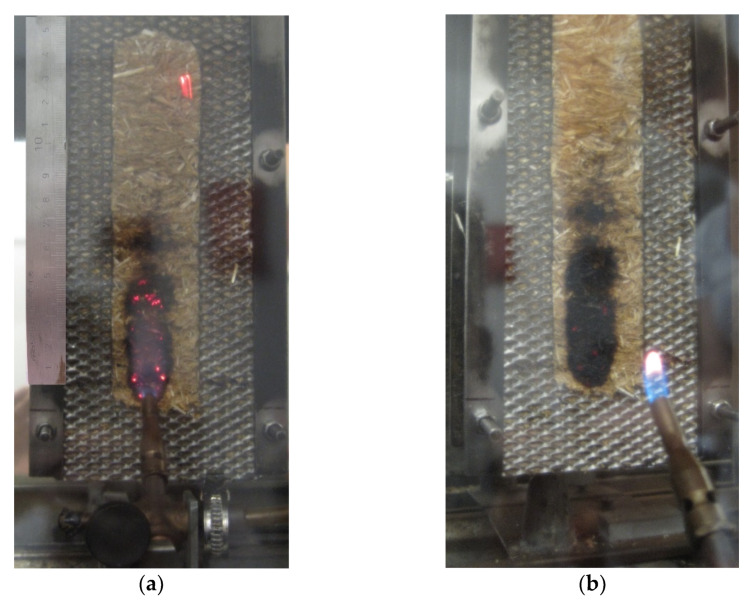
Crushed straw in direct exposure to flame (**a**); crushed straw after the ignitability fire test (**b**) [34].

The tested crushed straw was natural, without any chemical and artificial additives. The bulk density of the samples was 90 kg·m^−3^. The reason for choosing the bulk density of 90 kg/m^3^ was the finding that no settling would occur over time [18]. The sample was ignited, and after moving the burner away, the sample was immediately extinguished (see Figure 3a). The flame spread was up to 65 mm from the bottom edge (see Figure 3b). During the test, no burning particles of crushed straw fell off the container.

Experimental results are shown in Table 3. The single-flame source test showed that crushed straw placed in the structure does not heavily contribute to the spread of fire. Based on the results, crushed straw can be classified as a better fire reaction category than E. For this reason, another test was prepared: thermal attack by a single burning item.

### 4.2. Thermal Attack by a Single Burning Item Fire Test

**Figure 4:**
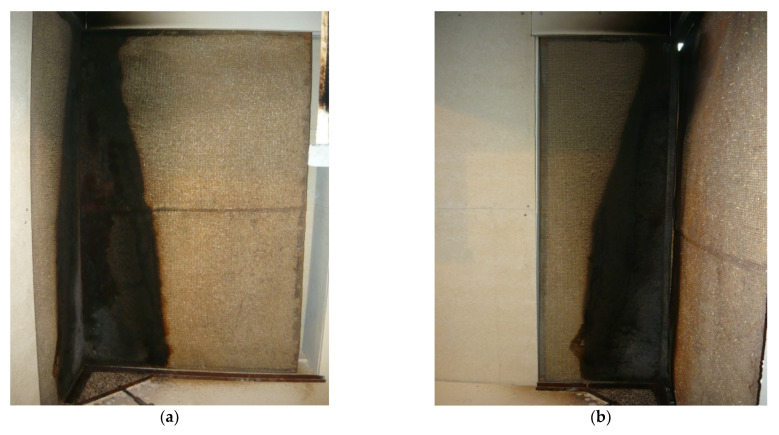
Long vertical wing of test sample No. 1 after the SBI test (**a**); short vertical wing of test sample No. 1 after SBI test (**b**).

During the SBI experiment at time t = (300 ± 5), the main burner ignited, and surface ignition of the test specimen occurred. The flame from the burner spread over the surface of the sample only to the upper edge of the test sample. There was no flame spread to the side edge of the short or longwing (see Figure 4). 

There was no such parameter LFS evaluation. No flaming particles or droplets fell from the surface of the exposed parts of the test sample. According to Table 1, the tested sample can be classified as d0. The smoke evolution rate parameter (SMOGRA) and the total smoke evolution from the test body release in the first 600 with a fire test (TSP600s) meeting the requirements for coating even in Class A2 according to the reaction to fire and crushed straw can be classified as s1 according to smoke spread. The fire spread index (FIGRA0.2MJ) exceeded the required value FIGRA0.2MJ ≤ 120 W/s, necessary for classification into reaction to fire class B. The difference between the required value and the measured value was very small. Crushed straw with a density of 90 kg·m^−3^ can be classified as Class C-s1, d0. The evaluation parameters and classification of the individual test samples from the experiment of crushed straw are given in Table 4.

### 4.3. Large-Scale Fire Test of a Wall Segment

**Figure 5:**
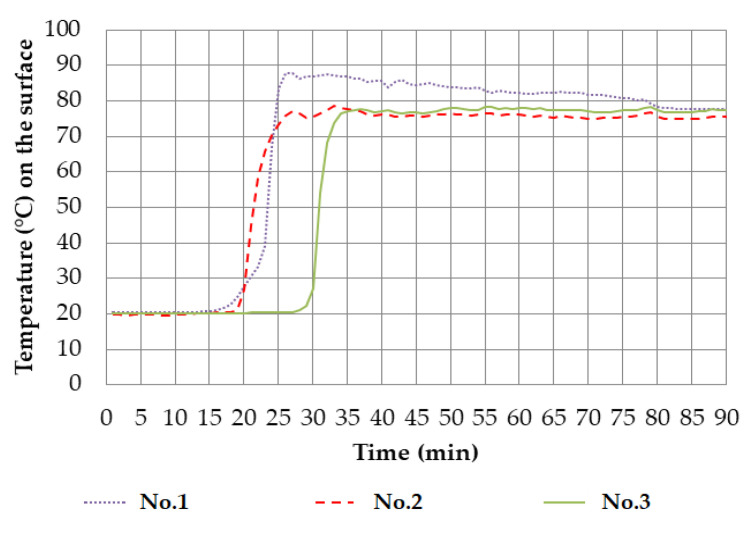
Curve of surface temperatures on the unexposed side of the test samples.

The fire test can be terminated after reaching one of the limit states (*E, I*) when the safety of operating personnel is endangered if there is a risk of damage to the test equipment or at the request of the client. In this case, the preliminary fire test was terminated when the integrity limit state was reached (*E)*. This limit state was reached in the 92nd minute from the start of the test. The insulation limit (*I*) was not reached during the test. The graphical temperature curve of all samples is shown in Figure 5. 

**Figure 6:**
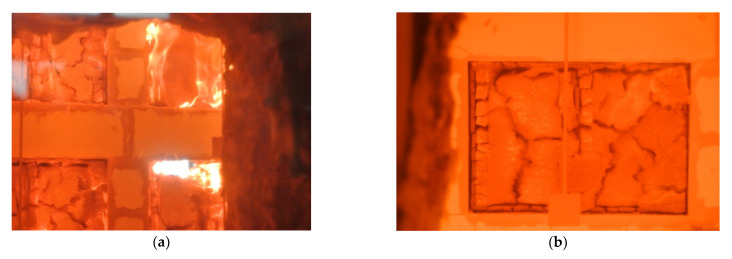
Exposed side of the test samples during the large-scale fire test in the tenth minute of the experiment. Front view of the burning samples (**a**); detailed view of sample No. 1 (**b**).

Sample No. 1 was coated on the exposed side with a 12.5 mm gypsum fibreboard, which itself reacts to the fire class of A2 (see Figure 6b). On the unexposed side, the cladding was made of 25 mm cement panel WS with reaction to fire in Class A2-s1. It was assumed that based on the cladding of the material classified as the better class of reaction to fire, the sample with this cladding will have the best fire resistance. This hypothesis was not confirmed. The best results from the tested sample were observed in the case of compositions with an oriented 15 mm strand board with reaction to fire class D on the exposed side (see Figure 6a).

From the measured values of surface temperatures on the unexposed side and the reaction of the tested samples in terms of the limit state of integrity (*E*) and insulation (*I*), a preliminary value of fire resistance *EI* (min) could be determined. As the test specimens were multilayered with wooden load-bearing elements suitable for wooden constructions, it is necessary to supplement the *EI* value with a classification of components and parts according to Categories DP1 (nonflammable structural system), DP2 (mixed structural system) and DP3 (flammable structural system). For this classification, the material from which the load-bearing part of the structure is made and its influence on the intensity of the fire are important. In Category DP1, the load-bearing system consists only of noncombustible materials (Classes A1, A2) and only for buildings up to a height of 2.5 m. Categories DP2 and DP3 include structures with load-bearing elements made of material with reaction to fire in Classes A2 (buildings higher than 2.5 m) to D. For their classification, however, the decisive material is the cladding of the structure, which is a reaction to fire class. This classification is important in practice because the legislation specifies exactly what materials can be used for a given space and function, e.g., load-bearing, fireproof, partition walls, horizontal structures, etc. This classification, according to structural parts, appears only in Czech legislation, as Czech requirements for wooden buildings are among the strictest in Europe. The tested segments contained load-bearing elements made of wood pertaining to reaction to fire class D. The cladding of the exposed side for sample No. 1 consisted of material with reaction to fire class A2, i.e., gypsum fibreboard FC. Therefore, the samples could be classified as Category DP2. For sample No. 2, the cladding on the exposed side consisted of a flammable OSB board of reaction to fire class D. This sample could only be classified into Class DP3. To determine the time of fire resistance of structures in category DP2, the decisive factor is the time for which one of the limit states and the integrity of the surface layers, the cladding, is reached. The surface layers must limit the burning of load-bearing parts and insulations (thermal or sound) so that they do not ignite in the required time, which could cause burning and increase the intensity of burning. The lowest time in which the first of the assessed limit states (*E, I, R*) was exceeded was taken as the determining time of fire resistance.

During the test, the time to reach the limit states *I* and *E* was monitored on the unexposed side of the furnace. Furthermore, the time for which the integrity of the cladding of the segments for classification into Categories DP2 and DP3 was ensured was monitored. After the sheathing fell off or burned off, the supporting elements of the samples burned out. In this case, the construction of warehouses identical to the samples cannot be classified into Category DP2. The crushed straw did not fall out during the test and was thus able, at least partly, to protect the supporting elements of the sample from the flame. In the case of load-bearing elements, only parts of them gradually burned out in places that were directly exposed to fire. The other elements were without major violations. Based on the measured surface temperatures on the unexposed side of the furnace and the behaviour of the samples during the fire test, the indicative values of fire resistance *EI* (min) were determined (see Table 5).

In the experiment, the temperature sensors were placed only on the unexposed surfaces of the samples, not inside. The estimation of the behaviour of the samples inside during the fire test was performed based on visual observation of the exposed side of the furnace and the measured surface temperatures. There was a rapid increase in the average surface temperature on the unexposed side after the sheathing burned off or fell off. Within 5 min between the 21st and 25th minutes, the surface temperature of the sample increased to the maximum surface temperature measured during the test (88 °C). Other samples had a similar course. It can be assumed that the course of fire propagation in samples with crushed straw after falling or burning of the casing will be identical to the course of flame propagation in the flammability test described in Section 4.1. Similarly, as in the single-flame source test, after the ignition of crushed straw and its charring in the sample, the flames did not spread deeper into the layer of crushed straw. This finding corresponds to the theory of flame nonpropagation in the straw to the depth.

Based on the results, one can notice that most specimens classified as DP3 have a fire resistance of 90 min and can be classified as EI 90 [41]. This fire resistance could be higher for some specimens, but the preliminary fire test was completed at 92 min. Only segment No. 2 has a fire-resistance value of EI 60, as a permanent flame appeared in this sample at the 89th minute of the test. The achieved value of the tested samples allows designing load-bearing structures of the same compositions up to the fourth degree of fire safety of the fire department.

## 5. Discussion

All these fire tests need to be taken in terms of the practical use of these wall compositions, which can be highly valued alternatives to other thermal insulation materials in residential structures [42]. It is necessary to analyse other properties of these materials, e.g., the thermal conductivity of crushed straw is λ = 0.045 W∙m^−1^∙K^−1^, while cellulose as an alternative blown insulation has values of about λ = 0.036 W∙m^−1^∙K^−1^ [43]. The values for other thermal insulation materials are as follows: wood λ = 0.2 W∙m^−1^∙K^−1^, gypsum board, λ = 0.17 W∙m^−1^∙K^−1^; rock wool, λ = 0.38 W∙m^−1^∙K^−1^ [44]. From this point of view, crushed straw is not the best, but it shows good performance.

In terms of fire resistance, straw has similar or the same properties as cellulose and, at the same time, much better resistance than mineral wool, fibreglass and bamboo, even by adding fire retardants [44,45]. From the point of view of the fire properties of crushed straw, i.e., reaction to fire and the fire resistance of structures filled with crushed straw, fire tests have shown that crushed straw has comparable fire properties to natural blown insulation used today. 

The requirement for fire resistance then depends on the type of structure (for example, ceiling, wall, roof), whether the structure is located on the underground or above-ground floor and according to the degree of fire safety of the fire section (type of fire zone). For small standard buildings, such as single-family homes, the standard requires a fire resistance of at least 15 min. If the tested sample reaches fire resistance during the test, for example EI 15DP2, we know that it is possible to use such a construction, for example in family houses [41].

The reaction to fire class of crushed straw with a bulk density of 90 kg·m^−3^ was classified based on the SBI fire test into Class *C*-*s*1, *d*0 materials with limited contribution to fire. For example, blown cellulose in the wall, with a bulk density of 70 kg·m^−3^ (trade name CLIMATIZER PLUS, CZ), reacts to fire *B*-*s*1, *d*0. Therefore, blown cellulose is a material with a very limited contribution to fire. Blown wooden fibre (trade name STEICO ZELL, CZ) with a bulk density of 32 to 40 kg·m^−3^ has a reaction to fire *B*-*s*2, *d*0. Both materials have a lower contribution to fire than crushed straw, but in terms of normative properties, all these materials are flammable. Because they are not classified as *A1* or *A2* (nonflammable materials), their use in building construction is limited. If we compare the results of the SBI fire test of crushed straw (see Table 4) with the classification criteria, we can see that crushed straw did not meet only the fire growth rate (FIGRA_0.2MJ_) criterion (W/s) [40]. The limit for classes A2 and B is FIGRA_0.2MJ_ ≤ 120 W/s. Crushed straw has FIGRA_0.2MJ_ = 123.0 to 134.9 W/s (see Table 4). For example, blown wooden fibre insulation (trade name STEICO ZELL) has FIGRA_0.2MJ_ ≤ 72.1 W/s. Blown cellulose insulation has FIGRA_0.2MJ_ about 100 W/s. If we compare other SBI fire test criteria, crushed straw has lower values than wooden and cellulose blown insulation. Improving the FIGRA criterion of crushed straw is possible only if we use the same chemical fire retardant; for example, if the cellulose insulation contains a magnesium sulphate fire retardant. However, for the practical use of crushed straw, flame retardants are of little use.

## 6. Conclusions

The presented research of the fire parameters of crushed straw has proven that crushed straw placed in a structure does not contribute to the spread of fire. The general assumption that constructions made of straw burn easily and quickly was thus dispelled. Crushed straw can be a suitable and cheaper alternative to other blown insulation. There is a necessity that crushed straw must be compacted to the required density in real construction. The minimum bulk density of crushed straw in structures should be 90 kg·m^−3^. Under these conditions, the flame acting on the surface of crushed straw does not get deeper into the structure, as the surface is partially closed. Thanks to this phenomenon, crushed straw can be used as insulation in wooden structures, to protect their load-bearing elements for a certain period, and thus to extend their fire resistance.

## Figures and Tables

**Table 1:** Results of thermal attack by single burning item classification parameters for Euro classes D to A2 [40].

Main Classification		Smoke Production		Flaming Droplets and Particles	
A2	FIGRA0.2 MJ ≤ 120 W/s LFS < sample edge THR600s ≤ 7.5 MJ	s1	SMOGRA ≤ 30 m^2^/s^2^ TSP600s ≤ 50 m^2^	d0	No flaming droplets/particles
B	FIGRA0.2 MJ ≤ 120 W/s LFS < sample edge THR600s ≤ 7.5 MJ	s1	SMOGRA ≤ 30 m^2^/s^2^ TSP600s ≤ 50 m^2^	d0	No flaming droplets/particles
C	FIGRA0.4 MJ ≤ 250 W/s LFS < sample edge THR600s ≤ 15 MJ	s2	SMOGRA ≤ 180 m^2^/s^2^ TSP600s ≤ 200 m^2^	d1	No flaming droplets/particles in EN 13823 persisting longer than 10 s within 600 s
D	FIGRA0 ≤ 750 W/s	s3	Not s1 or s2.	d2	Not d0 or d1

**Table 2:** Description of experimental samples of the preliminary large-format fire test [42].

Mark	Composition
1	Cement panel WS (25 mm) LAG frame (240 mm) Crushed straw (240 mm, 90 kg·m^−3^) Gypsum fibre boards FC (15 mm)
2	Wood fibre board (2 × 20 mm) LAG frame (240 mm) Crushed straw (240 mm, 90 kg·m^−3^) Oriented strand board (15 mm)
3	Wood fibre board (2 × 20 mm) LAG frame (240 mm) Crushed straw (240 mm, 90 kg·m^−3^) Gypsum fibre boards FC (15 mm)

**Table 3:** Results of the single-flame source test of crushed straw [34].

Parameter	Sample				
	1	2	3	4	5
Ignitability	Yes	Yes	Yes	Yes	Yes
Achieving the flame to the mark 150 mm	No	No	No	No	No
Burning time track 150 mm (s)	-	-	-	-	-

**Table 4:** SBI Test classification parameters and classification to Euro classes.

Classification Parameter	No. of the Test Sample			Mean
	1	2	3	
THR_600s_ (MJ)	5.2	5.6	5.4	5.4
LFS (Yes/No)	No	No	Ne	Ne
FIGRA_0.2MJ_ (W/s)	123.0	134.9	129.8	129.2
FIGRA_0.4MJ_ (W/s)	114.7	121.9	119.8	118.8
TSP600s (m^2^)	46.6	44.9	48.1	46.5
SMOGRA (m^2^/s^2^)	2.7	2.0	3.0	2.6
Reaction to fire	*C*-*s*1, *d*0	*C*-*s*1, *d*0	*C*-*s*1, *d*0	*C*-*s*1, *d*0

**Table 5:** Indicative values of fire resistance of the test segments EI (min).

Mark	Fire Resistance of the Test Segment for Category DP3	Fire Resistance of the Test Segment for Category DP2
1	EI 90 DP3	EI 15 DP2
2	EI 60 DP3	-
3	EI 90 DP3	EI 15 DP2

## Data Availability

All data are in the article.
